# Effect of TiO_2_ Calcination Pretreatment on the Performance of Pt/TiO_2_ Catalyst for CO Oxidation

**DOI:** 10.3390/molecules27123875

**Published:** 2022-06-16

**Authors:** Jianyu Cai, Zehui Yu, Xing Fan, Jian Li

**Affiliations:** Key Laboratory of Beijing on Regional Air Pollution Control, Beijing University of Technology, Beijing 100124, China; caijy@emails.bjut.edu.cn (J.C.); yuzehui@emails.bjut.edu.cn (Z.Y.); fanxing@bjut.edu.cn (X.F.)

**Keywords:** calcination pretreatment, Pt/TiO_2_, low temperature, CO oxidation, catalysis

## Abstract

In order to improve the CO catalytic oxidation performance of a Pt/TiO_2_ catalyst, a series of Pt/TiO_2_ catalysts were prepared via an impregnation method in this study, and various characterization methods were used to explore the effect of TiO_2_ calcination pretreatment on the CO catalytic oxidation performance of the catalysts. The results revealed that Pt/TiO_2_ (700 °C) prepared by TiO_2_ after calcination pretreatment at 700 °C exhibits a superior CO oxidation activity at low temperatures. After calcination pretreatment, the catalyst exhibited a suitable specific surface area and pore structure, which is beneficial to the diffusion of reactants and reaction products. At the same time, the proportion of adsorbed oxygen on the catalyst surface was increased, which promoted the oxidation of CO. After calcination pretreatment, the adsorption capacity of the catalyst for CO and CO_2_ decreased, which was beneficial for the simultaneous inhibition of the CO self-poisoning of Pt sites. In addition, the Pt species exhibited a higher degree of dispersion and a smaller particle size, thereby increasing the CO oxidation activity of the Pt/TiO_2_ (700 °C) catalyst.

## 1. Introduction

As one of the most effective methods for CO removal, CO catalytic oxidation is crucial for several practical applications [1,2,3]. Catalysts are key to the CO catalytic oxidation technology. CO catalysts mainly include precious metal catalysts [4,5,6,7] and non-precious metal catalysts [8,9,10]. Non-precious metal catalysts exhibit a good low-temperature activity, abundant resources, and cost-effectiveness, albeit with poor stability [11]. Although precious metal resources are scarce and expensive, they exhibit advantages of high CO oxidation performance and good stability. The further improvement in the low-temperature activity of precious metal catalysts, enhancement of catalyst stability, and reduction in the amount of precious metal are imperative to promoting practical applications of precious metal catalysts. Since the study on Pt-catalyzed CO oxidation by Langmuir [12], several researchers have conducted research on Pt catalysts [13,14,15,16,17,18].

Several studies have reported that TiO_2_-supported Pt catalysts exhibit high activity for CO oxidation [19,20]. Pt particle size [21,22], Pt dispersion [23], catalyst preparation method [24,25], type of catalyst carrier [26,27], use of doping additives [28,29], and catalyst pretreatment [30,31] mainly affect the CO oxidation performance of Pt catalysts. Bi [32] has prepared an N-doped N-Pt/TiO_2_ catalyst by flame spray pyrolysis, and investigated its CO oxidation performance. Pt-N bonds are formed on the catalyst surface, which change the ratio of oxygen species on the catalyst surface and the chemical state of Ti, which in turn improves the thermal stability of the catalyst. Jiang [33] has employed flame spray pyrolysis to introduce Au into the Pt/TiO_2_ catalyst to prepare a double precious metal catalyst of Au-Pt/TiO_2_. Au and Pt exist as alloys. The synergistic effect between Au and Pt reduces the agglomeration of noble metals and inhibits CO poisoning. The CO oxidation performance of the dual noble metal catalyst is 20% greater than that of the Pt/TiO_2_ catalyst. Mohamed [34] has compared the CO oxidation performance of a Pt/TiO_2_ catalyst prepared by impregnation and precipitation. He reported that the preparation method affects catalytic performance and that the Pt/TiO_2_ catalyst prepared by precipitation exhibits better low-temperature activity. Choi [35] has prepared a Pt/TiO_2_ catalyst by the aerogel method and reported that the catalyst exhibits a higher specific surface area. After calcination at 500 °C, the ratio of anatase type TiO_2_ and rutile type TiO_2_ are 90.2% and 9.8%, respectively. The catalyst exhibits a good low-temperature activity, with an activation energy of only 13.4 kcal·mol^−1^.

The modification method used in the above-mentioned research is relatively cumbersome. In order to explore a simple, environmentally friendly and efficient modification method, a series of Pt/TiO_2_ catalysts were prepared by impregnation, and their CO catalytic oxidation performance was investigated, as well as the effect of TiO_2_ calcination pretreatment on the performance of the Pt/TiO_2_ catalyst.

## 2. Experiments

### 2.1. TiO_2_ Pretreatment

TiO_2_ (China National Pharmaceutical Group Corporation, Beijing, China) was roasted for 4 h in a muffle furnace (Tianjin Zhonghuan Furnace Corp, Tianjin, China) at 600 °C, 650 °C, 700 °C, 750 °C, and 800 °C.

### 2.2. Catalyst Preparation

Pt/TiO_2_ catalysts were prepared by impregnation and the mass fraction of Pt was 0.5%. Specific steps were as follows: First, TiO_2_ was added to an aqueous solution of H_2_PtCl_6_ (China National Pharmaceutical Group Corporation, Beijing, China), and the resulting suspension was ultrasonically stirred at 50 °C for 3 h in a water bath. After impregnation, the suspension was dried at 110 °C in a blast drying oven (Tianjin Zhonghuan Furnace Corp, Tianjin, China) and subsequently treated at 450 °C for 3 h, affording a Pt/TiO_2_ catalyst. The catalysts prepared by TiO_2_ subjected to calcination at different temperatures are expressed as Pt/TiO_2_ (600 °C), Pt/TiO_2_ (650 °C), Pt/TiO_2_ (700 °C), Pt/TiO_2_ (750 °C), and Pt/TiO_2_ (800 °C). The mass fraction of Pt in all catalysts was 0.5%. In order to distinguish different catalysts, the catalysts without carrier calcination pretreatment are named Pt/TiO_2_, and the catalysts obtained by carrier calcination pretreatment at different temperatures are collectively referred to as Pt/TiO_2_ (M°C).

### 2.3. Catalyst Characterization

The surface areas of the catalysts were determined by the BET specific surface area test method using a Micromeritics Gemini V instrument (Norcross, GA, USA). XRD patterns of the catalysts were recorded on a Bruker D8 Advance instrument (Karlsruhe, Germany) operated at 40 kV and 40 mA using nickel-filtered Cu Kα radiation (λ = 0.15406 nm). Catalyst morphology was observed by a Crossbeam 350 SEM instrument (Oberkochen, Germany) operating at 5 kV, and the samples were coated with gold for 30 s before measurement. Surface chemical states of the Pt/TiO_2_ catalysts were investigated by XPS (ESCALAB 250Xi, Waltham, MA, United Kingdom) using an Al Kα X-ray source (1486.7 eV) at 15 kV and 25 W, with the binding energy calibrated by C1s at 284.8 eV. CO chemisorption experiments were conducted as follows: First, 100 mg of a sample (40–60 mesh) was reduced under 10% H_2_ for 1 h, followed by cooling to 40 °C and purging with argon for 15 min. Subsequently, a pulse adsorption test using a 10% CO-He mixture was conducted. To investigate the adsorption performance of the catalyst for CO, the CO-TPD test was conducted using a BELCAT-B instrument (Osaka, Japan). The test method is as follows: First, 100 mg of a sample (40–60 mesh) was pretreated for 30 min under He at 300 °C. After cooling to 25 °C, CO gas was injected at a gas volume of 50 mL/min for 60 min. Then, it was purged with He at 25 °C for 30 min, and finally heated to 700 °C at a heating rate of 5 °C/min. The desorbed gas component was detected by a mass spectrometer.

### 2.4. Catalytic Testing

The catalytic test was conducted in a continuous-flow fixed-bed quartz reactor using 1.8760 g of the catalyst at a total gas flow rate of 90,000 cm^3^/h, a simulated flue gas of 10,000 mg/m^3^ CO, 16% O_2_ and balanced by N_2_. An MRU infrared flue gas analyzer (MGA6 Plus, MRU, Obereisesheim, Germany) was used to monitor the CO concentration at the outlet. The CO removal efficiency was calculated by the following equation:

(1) $$\eta =\frac{\left[CO_{in}\right]-[CO_{out}]}{\left[CO_{in}\right]}\times 100\%\;$$

where $\left[CO_{in}\right]$ is the inlet CO content of the catalyst and $\left[CO_{out}\right]$ is the outlet CO content of the catalyst.

In addition, the sulfur and water resistance of the catalyst was evaluated using the above device by the addition of 15% water vapor and 0.005% SO_2_ to the simulated flue gas at 190 °C.

## 3. Results and Discussion

### 3.1. Catalytic Performance

**Figure 1:**
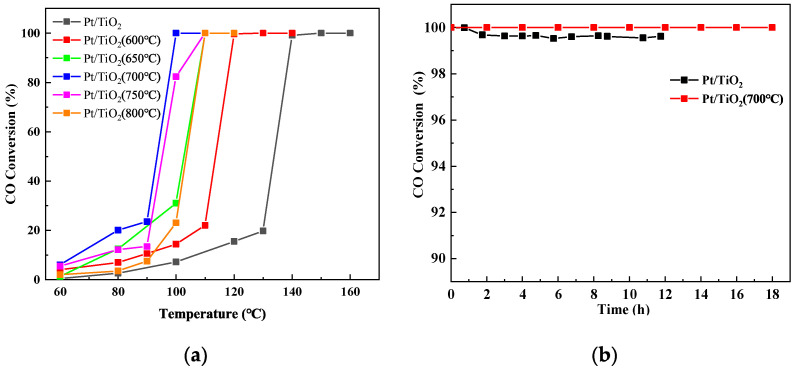
(**a**) Activity of the catalytic oxidation of CO. (**b**) Activity of the catalytic oxidation of CO in the presence of SO_2_ and water vapor.

Different Pt/TiO_2_ catalysts were investigated for their CO oxidation performance. Figure 1a shows the results. With the increase in temperature, the CO conversion for all catalysts for CO increase. After the calcination and pretreatment of TiO_2_, the CO oxidation performance of the Pt/TiO_2_ catalyst is greater than that of the Pt/TiO_2_ catalyst without pretreatment. With the increase in the calcination pretreatment temperature, the CO oxidation performance of the Pt/TiO_2_ catalyst exhibits an increase first and then a decrease; the temperature of the complete conversion first decreases, then increases with the increase in the calcination temperature. With the calcination pretreatment temperature reaching 700 °C, the highest CO oxidation performance is observed. When the temperature reaches 100 °C, the removal efficiency of CO by Pt/TiO_2_ (700 °C) can reach 100%, while the removal efficiency of CO by Pt/TiO_2_ is less than 10%. After calcination pretreatment at 700 °C, the complete conversion temperature of CO is reduced by 40 °C.

When sintering flue gas, chemical plant exhaust gas and other actual flue gas often contain SO_2_ and H_2_O. Therefore, the sulfur and water resistance properties of the catalyst are very important. It is of great significance for the practical application of the catalyst to investigate the effect of the calcination pretreatment on the performance of the catalyst. Figure 1b shows the sulfur and water resistance experiment of Pt/TiO_2_ and Pt/TiO_2_ (700 °C) catalysts. By continuous testing at 190 °C for 18 h, the CO removal efficiency of Pt/TiO_2_ (700 °C) was maintained at 100%, and CO was not detected at the outlet. However, after 12 h of continuous testing, the CO removal efficiency of Pt/TiO_2_ dropped to about 99.6%. The results revealed that the Pt/TiO_2_ (700 °C) catalyst exhibits good stability under the experimental conditions. In order to explore the reasons for the effect of support calcination pretreatment on the catalyst activity and stability, a series of characterization analyses were carried out on the catalyst.

### 3.2. Catalyst Characterization

#### 3.2.1. BET

**Figure 2:**
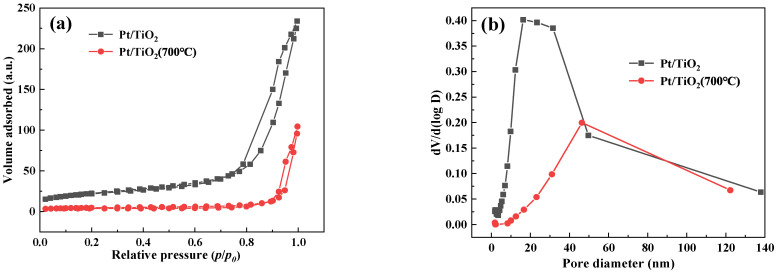
(**a**) N_2_ adsorption–desorption isotherms; (**b**) Pore-size distributions of catalysts.

To investigate the effect of calcination pretreatment on Pt/TiO_2_ and Pt/TiO_2_ (700 °C), their physical structure characteristics were examined. Figure 2a is the N_2_ adsorption and desorption isotherm of Pt/TiO_2_ and Pt/TiO_2_ (700 °C) catalysts. Both catalysts showed type IV adsorption and desorption curves, and a H3 lag loop appeared under the relative pressure (P/P0) from 0.8 to 1.0 and 0.9 to 1.0, respectively, indicating that both catalysts had mesoporous structures. Comparing the two curves in Figure 2b, it is found that the pore size of catalyst Pt/TiO_2_ is mostly distributed around 20 nm, while the pore size of catalyst Pt/TiO_2_ (700 °C) is mostly distributed around 50 nm. Table 1 is the physical structure characteristics data, and Pt/TiO_2_-SH and Pt/TiO_2_ (700 °C)-SH represent the samples of Pt/TiO_2_ and Pt/TiO_2_ (700 °C) after the sulfur and water resistance experiment, respectively. The BET surface area and pore volume of Pt/TiO_2_ (700 °C) exhibit a decreasing trend; however, the pore size increases significantly. A larger pore size facilitates diffusion of reactants and reaction products. The results show that the specific surface area, pore volume and pore size of the catalyst exhibit a downward trend after the sulfur and water resistance test, as shown at the end of Table 1 (Pt/TiO_2_-SH and Pt/TiO_2_ (700 °C)-SH). The reason for this result may be the accumulation of sulfate on the catalyst surface.

#### 3.2.2. XRD

**Figure 3:**
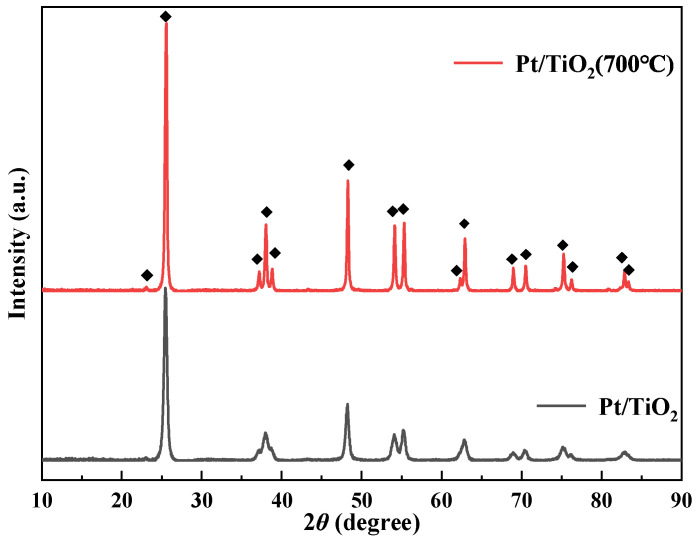
XRD patterns of catalysts.

To investigate the effect of calcination pretreatment on the crystal form of Pt/TiO_2_, XRD analysis was conducted. Figure 3 shows the XRD patterns of catalysts recorded at a 2*θ* range of 20–90°. Pt/TiO_2_ and Pt/TiO_2_ (700 °C) exhibit typical anatase TiO_2_ diffraction peaks at 2*θ* = 25.3°, 37.0°, 37.5°, 37.8°, 48°, 53.9°, 55°, 62.7°, 68.8°, 70.5°, 75.1°, and 76.1° (JCPDS No.21-1272). The results revealed that after the calcination pretreatment temperature reaches 700 °C, the crystal phase of the catalyst did not change. Comparing the XRD patterns of the two catalysts, it is found that the crystallinity of the Pt/TiO_2_ (700 °C) catalyst is significantly improved. The improvement of catalyst crystallinity will reduce the content of amorphous substances in the pores, promoting the diffusion of reactants and reaction products, and is beneficial to the improvement of catalyst activity. A characteristic peak of Pt is absent in the XRD test result, indicating that Pt is highly dispersed in TiO_2_.

#### 3.2.3. SEM

**Figure 4:**
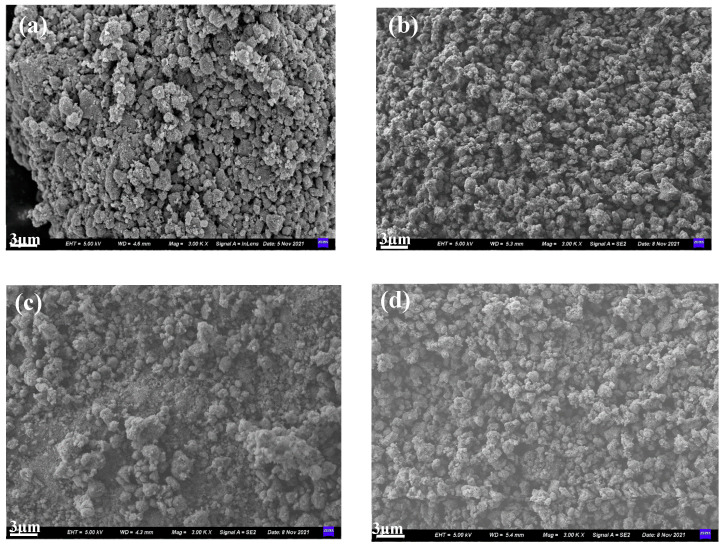
SEM images of (**a**) Pt/TiO_2_, (**b**) Pt/TiO_2_ (700 °C), (**c**) Pt/TiO_2_-SH and (**d**) Pt/TiO_2_ (700 °C)-SH catalysts.

SEM was employed to investigate the effect of calcination pretreatment on the surface structure and morphology of the catalysts; Figure 4 shows the results. Pt/TiO_2_-SH and Pt/TiO_2_ (700 °C)-SH represent Pt/TiO_2_ and Pt/TiO_2_ (700 °C) after the sulfur and water resistance test, respectively. The SEM image (a) shows that the particles on the Pt/TiO_2_ surface vary in size. However, the SEM image (b) shows that the particles on the surface of Pt/TiO_2_ (700 °C) are fine and uniform. The comparison of the SEM images (a) and (b) revealed that after calcination at 700 °C, the surface roughness of TiO_2_ particles is reduced, and the particle size and pore distribution are uniform. Such a surface may be beneficial for the effective loading of Pt. The SEM images of Pt/TiO_2_ (700 °C) and Pt/TiO_2_ (700 °C)-SH revealed that Pt/TiO_2_ (700 °C) changes significantly before and after the sulfur and water resistance test. The Pt/TiO_2_ (700 °C)-SH surface is covered by flocs, and the pores are blocked, inferring that after the sulfur and water resistance test of Pt/TiO_2_ (700 °C), sulfate is formed on the surface, which covers the catalyst surface.

#### 3.2.4. XPS

**Figure 5:**
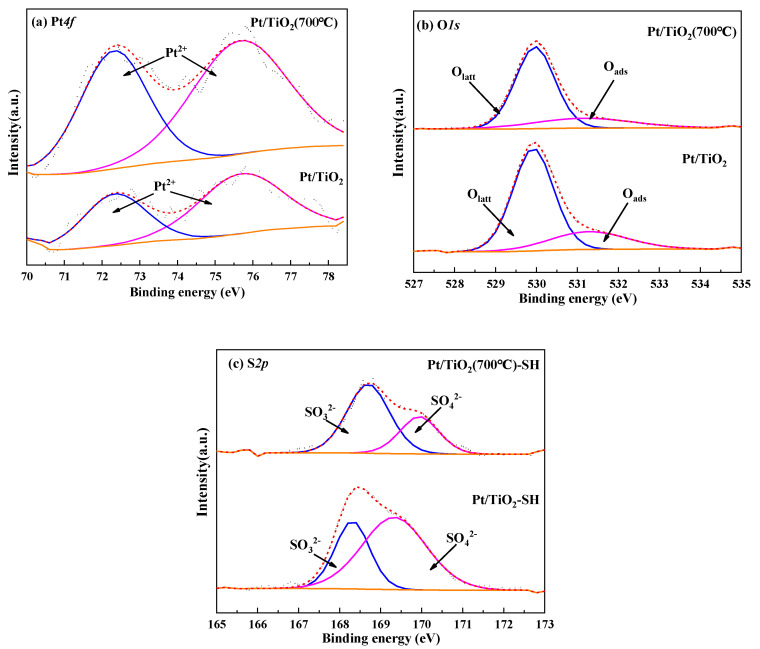
XPS profiles for catalysts.

The chemical state of the elements on the catalysts surface was analyzed by XPS. Figure 5 shows the XPS profiles, and the surface element compositions are shown in Table 2. Looking at Figure 5a,b, Pt on the surface of the two catalysts is present in PtO and the surfaces of both catalysts contain two oxygen elements; the peaks around 530 eV and 531.6 eV are assigned to lattice oxygen (Olatt) and adsorbed oxygen (Oads), respectively [36]. Table 2 shows that the proportion of O_ads_ on the surface of Pt/TiO_2_ (700 °C) is significantly higher than that of Pt/TiO_2_, which will be beneficial to the improvement of the CO oxidation activity of Pt/TiO_2_ (700°C). In addition, Figure 5c shows that S element will accumulate on the surface of the catalyst after sulfur and water resistance experiments, and it exists in the form of SO_3_^2−^ and SO_4_^2−^. This result indicated that under the condition of SO_2_ and H_2_O, sulfate is formed on the catalyst surface. This result is consistent with those reported by Taira [37]. SO_2_ and H_2_O can form TiOSO_4_ on the TiO_2_ surface. However, from the viewpoint of catalytic efficiency, the formation of sulfate does not affect the CO oxidation activity of the Pt/TiO_2_ (700 °C) catalyst, probably because SO_2_ inhibits the catalytic performance of CO, H_2_O can promote the oxidation of CO and the promotion effect of H_2_O is greater than the inhibition effect of SO_2_ [38]. By comparing the proportion of SO_3_^2−^ on the surface of the two catalysts, it is found that the proportion of SO_3_^2−^ on the surface of Pt/TiO_2_ (700 °C)-SH is significantly higher than that of Pt/TiO_2_-SH, while SO_3_^2−^ is more unstable and easier to decompose, which will be beneficial to the regeneration of Pt/TiO_2_ (700 °C)-SH.

#### 3.2.5. CO Chemisorption Experiments

The dispersion and particle size of precious metals affect catalyst performance. To explore the effect of the pre-calcination of the carrier on the catalyst performance, CO chemisorption tests were conducted on the catalyst. The adsorption amount of CO on the catalyst is obtained by CO pulse adsorption and then converted into the number of adsorbed CO atoms (N_co_). According to the content of Pt in the catalyst, the number of Pt atoms in the catalyst (N_Pt_) can be obtained, and the dispersion of Pt can be obtained by N_co_/N_Pt_. As can be seen in Table 3, the dispersions of Pt species are 52.44% and 60.27% on the Pt/TiO_2_ and Pt/TiO_2_ (700 °C) catalysts, respectively. Furthermore, the Pt particle size of Pt/TiO_2_ is 18.01 nm, in comparison with 15.67 nm observed for Pt/TiO_2_ (700 °C). Calcination pretreatment of the carrier can improve the dispersion of precious metals and reduce the particle size of Pt, thereby improving the CO performance of the catalyst.

#### 3.2.6. CO-TPD

**Figure 6:**
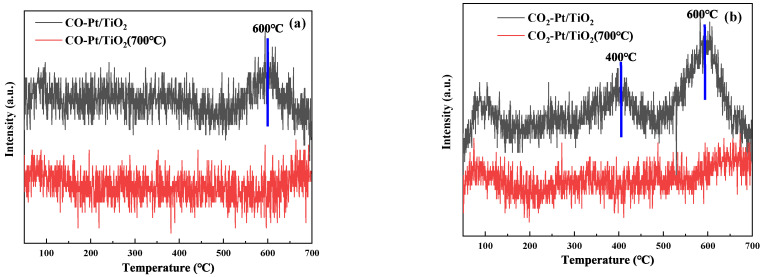
CO-TPD test results of two catalysts: (**a**) CO test results; (**b**) CO_2_ test results.

To compare the adsorption and desorption performance of the two catalysts for CO, a CO-TPD test was conducted, and the desorption gas was examined by mass spectrometry. Desorption curves of CO and CO_2_ in the two catalysts were obtained. Figure 6a,b shows the test results. Although the CO adsorption capacity of the two catalysts is extremely low, careful comparison of the desorption peaks of CO and CO_2_ revealed that the desorption of CO and CO_2_ in the Pt/TiO_2_ (700 °C) catalyst is less than that of the Pt/TiO_2_ catalyst. Due to the low adsorption capacity of CO on the two catalysts, the noise interference of the TPD curve is serious. The most obvious peak in the figure can be analyzed, in which it is difficult to observe the CO desorption peak on the Pt/TiO_2_ (700 °C) catalyst, but a more obvious peak is observed on the Pt/TiO_2_ catalyst at about 600 °C. In addition, the desorption peak of CO_2_ on the Pt/TiO_2_ (700 °C) catalyst is weak, and the Pt/TiO_2_ catalyst has more obvious peaks at about 400 °C and 600 °C. This result implies that a low amount of CO is absorbed on the Pt/TiO_2_ (700 °C) catalyst, which inhibits the adsorption of CO by Pt, alleviates the self-poisoning phenomenon of the Pt catalyst, and promotes the low-temperature catalytic effect of Pt.

## 4. Conclusions

In this study, a simple, environmentally friendly and efficient method for catalyst modification was explored. This study revealed that the calcination pretreatment of the carrier helps to optimize the specific surface area and pore structure of the carrier, which is beneficial to the diffusion of reactants and reactants. At the same time, the proportion of adsorbed oxygen on the catalyst surface increased, promoting the oxidation of CO. After the carrier was pretreated by calcination, the adsorption capacity of the catalyst for CO and CO_2_ was reduced, facilitating the diffusion of CO and desorption of CO_2_, reducing the occupation of active sites, and promoting the reaction. Reducing the CO adsorption capacity effectively suppressed the CO self-poisoning phenomenon of Pt and improved the low-temperature activity of the catalyst. In addition, the pretreatment of the calcined carrier improved the dispersion of Pt species and reduced the Pt particle size. As a result, the CO oxidation activity of the catalyst was improved, and the complete conversion of CO was realized at 100 °C, 40 °C less than that of the unpretreated sample.

## Figures and Tables

**Table 1:** Physical structure characteristics data.

Catalyst	BET Surface Area m^2^·g^−1^	Pore Volume cm^3^·g^−1^	Pore Size nm
Pt/TiO_2_	78.78	0.363	17.0
Pt/TiO_2_ (700 °C)	15.76	0.155	42.9
Pt/TiO_2_-SH	76.50	0.354	15.1
Pt/TiO_2_ (700 °C)-SH	15.21	0.151	41.9

**Table 2:** Surface element compositions of catalysts.

Catalyst	$\frac{O_{ads}}{O_{ads}+O_{latt}}$	$\frac{SO_{3}^{2-}}{SO_{3}^{2-}+SO_{4}^{2-}}$
Pt/TiO_2_	0.206	/
Pt/TiO_2_ (700 °C)	0.295	/
Pt/TiO_2_-SH	/	0.369
Pt/TiO_2_ (700 °C)-SH	/	0.669

**Table 3:** Platinum dispersion, platinum particle size, and platinum surface area of catalysts determined by CO chemisorption.

Catalyst	Platinum Dispersion %	Platinum Particle Size nm	Platinum Surface Area m^2^·g^−1^
Pt/TiO_2_	52.44	18.01	0.26
Pt/TiO_2_ (700 °C)	60.27	15.67	0.92

## Data Availability

Not applicable.

## Sample Availability

Samples of the compounds Pt/TiO_2_ and Pt/TiO_2_(M°C) are available from the authors.
